# 282. Risk Factors for Mortality in Severe COVID-19 Patients Admitted to the Intensive Care Unit: A Retrospective Single-Center Study in Saudi Arabia

**DOI:** 10.1093/ofid/ofab466.484

**Published:** 2021-12-04

**Authors:** Sherif Khattab, Souad AlMuthree, Mohamed Bakry, Noha Ibraheem, Omar Alghamdi, Mahassen Khalifa, Ibrahim Alsehli, Paul McCague

**Affiliations:** 1 Queen’s University Belfast, Dubai, Dubai, United Arab Emirates; 2 King Abdullah Medical City, Makkah, Saudi Arabia, Makkah, Makkah, Saudi Arabia; 3 Biostatistics, Dubai, Dubai, United Arab Emirates

## Abstract

**Background:**

The first case of COVID-19 in the Kingdom of Saudi Arabia (KSA) was reported in March 2020. This study aims to describe the overall mortality in the ICU during the COVID-19 pandemic and to determine independent risk factors for overall survival & 29 days mortality.

**Methods:**

This is a retrospective single-center study; data for adult patients admitted to the ICU with COVID-19 between 1^st^ March 2020 to 31^st^ December 2020 were extracted and reviewed. Overall survival was described using Kaplan-Meier curves with reporting of median overall survival and 29 days survival estimates. Multivariate analysis was performed using Cox proportional hazards model and multivariate logistic regression model.

Figure 1. Study flow chart

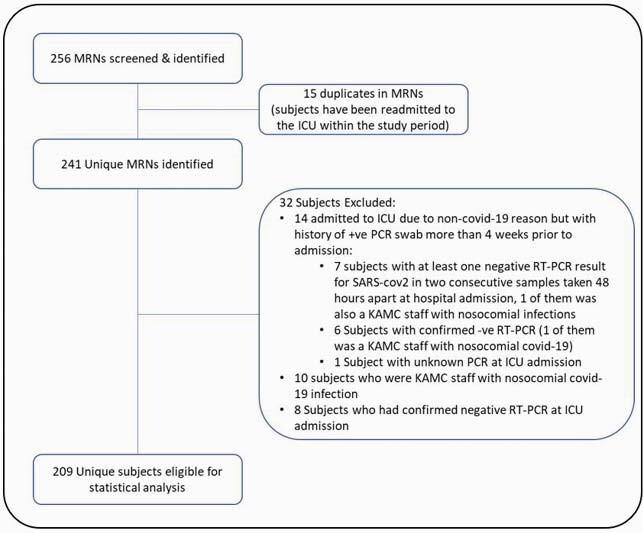

Table 1. Demographic characteristics categorized by Gender

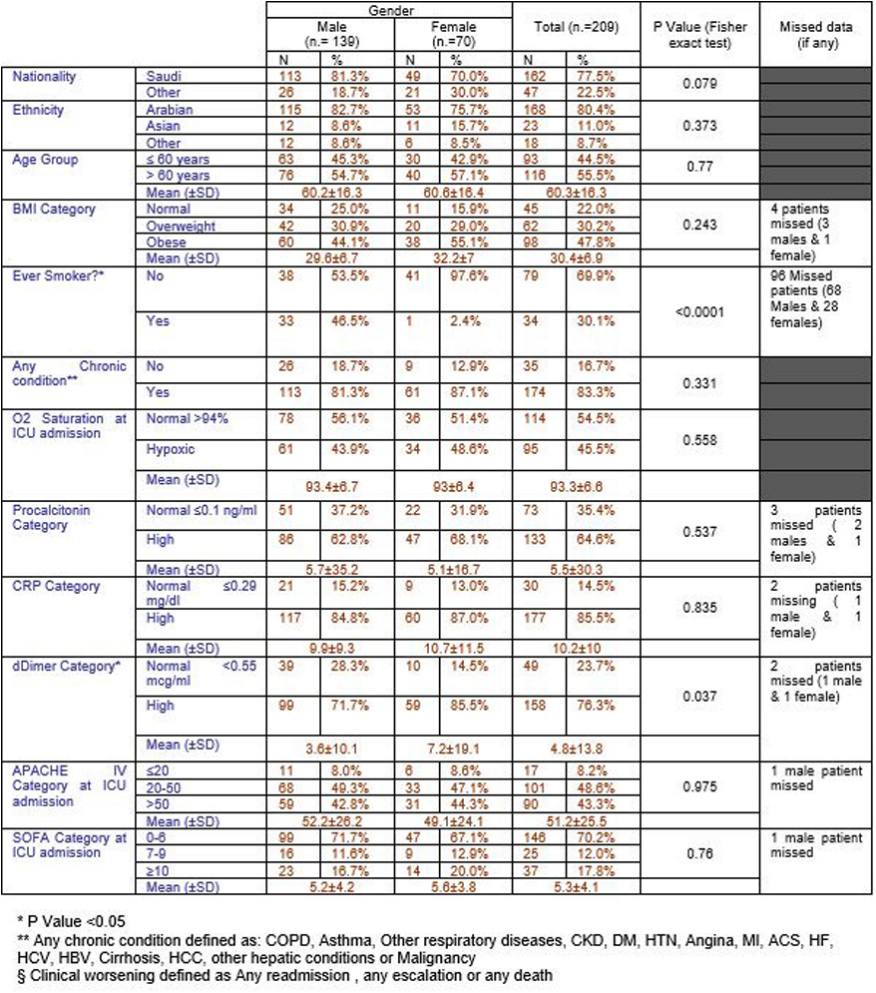

**Results:**

Eligible subjects were 209 (Figure 1) and subjects demographics are summarized in (Table1). Observed death events were 82 (39.2% of the total cohort), 61% of deaths reached at 2 weeks of ICU stay (n.= 50), median overall survival (OS) was reached at day 23, as shown in (Figure 2). The multivariate Cox proportional hazard regression analysis (Figure 3) showed elevated SOFA score [aHR= 1.10, P < 0.001] and Vasopressors [aHR= 3.23, P= 0.002] as independent risk factors for overall ICU mortality. Independent protective factors were: Systemic corticosteroids use (P= 0.019), Insulin use (P= 0.026) and Liposomal Amphotericin B (LAMB) use (P= 0.019). For mortality at day 29, the multivariate logistic regression model (Figure. 4) showed elevated SOFA score (P= 0.005), any need for ventilation escalation after ICU admission (P= 0.014), Ribavirin use (P=0.016) and Vasopressors use ( P< 0.001) as independent risk factors. Angiotensin-Converting Enzyme inhibitors (ACEi) use was a protective factor (P=0.025).

Figure 2. Overall Survival (OS) for patients admitted to the ICU due to COVID-19 - Kaplan Meier (KM)

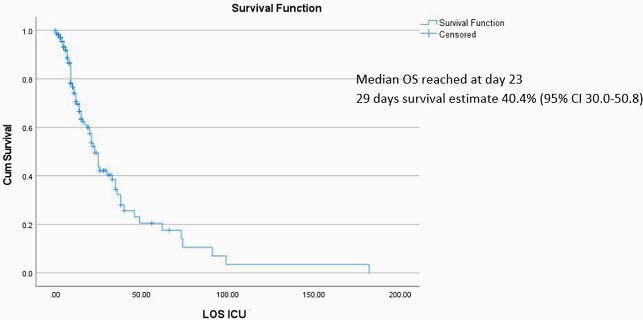

Figure 3. Multivariate Cox proportional hazard regression model for factors associated with overall mortality in patients admitted to the ICU due to COVID-19

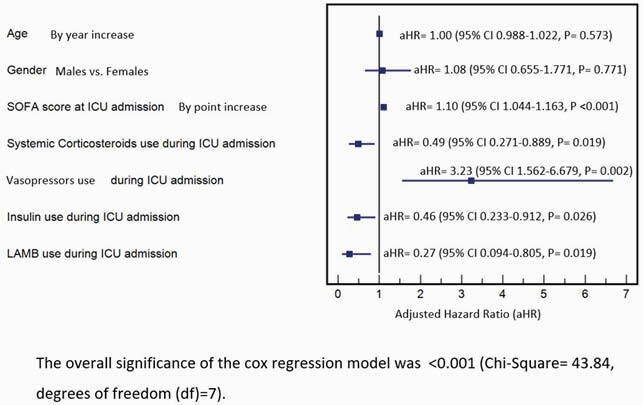

Figure 4. Multivariate logistic regression model for factors associated with 29 days mortality in patients admitted to the ICU due to COVID-19

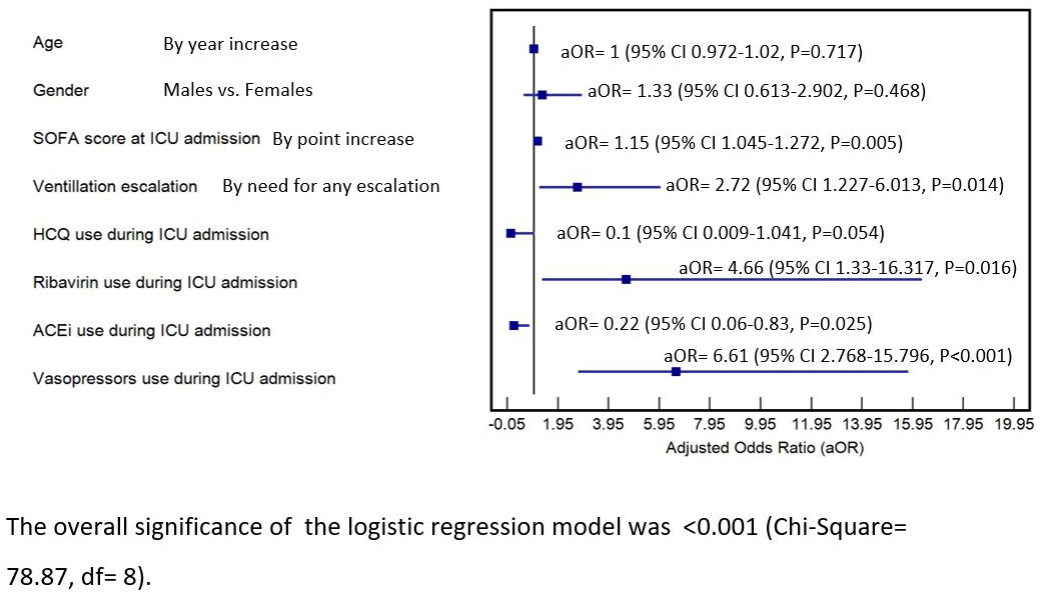

**Conclusion:**

SOFA score and vasopressors are independent predictors for overall survival and 29-day mortality in the ICU. The need for ventilation escalation after ICU admission appeared to lead to poor prognosis in regard to 29-day mortality only. Systemic corticosteroids are lifesaving, further studies are required to confirm the observed clinical benefits with insulin, LAMB and ACEi use in the ICU and to investigate any hazardous impact of ribavirin on COVID-19 outcomes.

**Study limitations:**

Residual confounding of other measured and/or unobserved factors cannot be ruled out.

**Disclosures:**

**Sherif Khattab, BPharm**, **Gilead Sciences** (Employee, Shareholder) **Mohamed Bakry, MBBCh**, **Gilead Sciences** (Employee)**Roche Pharma** (Employee)

